# The Role of 4G Allele in Plasminogen Activator Inhibitor-1 (rs1799889) Gene as Biomarker for Thrombophilic States among Patients with Type 2 Diabetes: Plasminogen Activator Inhibitor-1 4G Allele for Thrombophilic States of Type 2 Diabetes

**DOI:** 10.31661/gmj.v11i.2447

**Published:** 2022-08-03

**Authors:** Rowida Eljack Ibrahim, Khalid Abdelsamea Mohamedahmed

**Affiliations:** ^1^ Department of Hematology, Faculty of Medical Laboratory Sciences, University of Gezira, Wad Medani, Sudan; ^2^ Department of Immunology, Faculty of Medical Laboratory Sciences, University of Gezira, Wad Medani, Sudan

**Keywords:** 4G Allele, lasminogen Activator Inhibitor 1, rs1799889, Thrombosis, Diabetes Mellitus

## Dear Editor,

Diabetes is a group of metabolic disorders characterized and identified by the presence of hyperglycemia in the absence of treatment. Two main forms of diabetes mellitus (DM) are distinguished; type 1 and type 2. Type 2 DM, previously known as non-insulin-dependent, is more prevalent and responsible for 90% of the disease prevalence [1]. It is among the top 10 causes of death in adults and was estimated to have caused four million deaths globally in 2017 [2].

Type 2 DM can lead to microvascular (i.e., neuropathy, nephropathy, and retinopathy) and macrovascular (i.e., coronary artery disease, stroke, and peripheral vascular disease) complications, which are associated with an increased risk of atherosclerosis and thrombosis that are major causes of death in 80% of patients with DM. Of these deaths, more than 75% are due to cardiovascular complications, while the remaining is due to cerebrovascular events and peripheral vascular complications [3].

Chronic hyperglycemia is not the only cause of these complications; these ischemic events are also associated with platelet hyperactivation, abnormal activation of coagulation proteins, abnormal endothelial function, and hypo-fibrinolysis [4].

Also, patients with type 2 DM present hypercoagulability and hypofibrinolysis. The imbalance between coagulation and fibrinolysis mainly contributes to excess fibrin deposition in the vascular wall (due to hypofibrinolysis) and then results in the pathogenesis of atherothrombosis. Hypofibrinolysis leads to thrombotic events and represents one of the major causes of morbidity, mortality, and high healthcare costs [5]. The hypofibrinolysis occurs mainly by elevated plasma concentrations of plasminogen activator inhibitor (PAI)-1, a cornerstone of fibrinolysis regulation. Overexpression of PAI-1 can be affected by genetic and metabolic factors; higher PAI-1 levels decrease fibrinolysis and promote atherothrombosis [6].

The PAI-1 gene is located on human chromosome 7q21.3-22 with eight introns and nine exons spanning 12.3 kb. It belongs to the serine protease inhibitor (serpin) family and is the main physiological inhibitor of tissue-type plasminogen activator (tPA) in the fibrinolytic system, which produces active plasmin from plasminogen that then cleaves fibrin [7].

Impaired fibrinolytic function induced by increased PAI-1 expression is commonly observed in patients with thrombotic disease [8], and different PAI-1 polymorphisms induced the increase of its level described previously by various researchers. The most commonly described till now is the PAI-1 (rs1799889)-675 4G/5G insertion/deletion polymorphism at -675 in the promoter region [9].

This polymorphism produces two alleles that contain either four or five sequential guanosines (4G and 5G) that differ in their regulation of the concentration of PAl-1 [10]. Subjects who are homozygous for the 4G allele have plasma PAI-1 concentrations approximately 25% higher than those of subjects who are homozygous for the 5G allele. The specific genotype of PAI-1 interacts with some metabolic factors (such as plasma triglyceride, high-density lipoprotein, plasma insulin, visceral fat, circumference width, and body mass index), which are contributed to increasing the plasma concentration of PAI-1 and therefore possibly have an increased risk for intravascular thrombosis and recurrent myocardial infarction (MI) in young patients with DM [5][6].

Previously, most researchers identified the allele 4G of the PAI-1 (rs1799889) as an independent risk factor for MI in young individuals, and PAI-1 plasma concentrations for the individuals' carrier 4G allele were higher by 25% more than the 5G allele (4G allele transcribe of PAI-1 six times more than 5G allele) [6].

We concluded that the molecular diagnosis of the 4G allele and their level are good enough to evaluate the impairment of fibrinolysis in patients with type 2 DM. Furthermore, the patients with type 2 DM positive for the 4G allele must be given antithrombotic drugs like aspirin at least as the first-line protection from thrombus formation, especially in cardiovascular events because when the platelet is active release about 90% of the PAI-1 Ag.

## Conflict of Interest

The authors have declared that no competing interests exist.
